# Four new species of Desmiphorini (Coleoptera, Cerambycidae, Lamiinae)

**DOI:** 10.3897/zookeys.513.9947

**Published:** 2015-07-15

**Authors:** Ubirajara R. Martins, Maria Helena M. Galileo, Antonio Santos-Silva

**Affiliations:** 1 Museu de Zoologia, Universidade de São Paulo, São Paulo, São Paulo, Brazil (researcher of CNPq); 2 PPG Biologia Animal, Departamento de Zoologia, Universidade Federal do Rio Grande do Sul, Porto Alegre, RS, Brazil (Fellow of the Conselho Nacional de Desenvolvimento Científico e Tecnológico); 3 Museu de Zoologia, Universidade de São Paulo, São Paulo, São Paulo, Brazil

**Keywords:** Belize, Bolivia, Taxonomy

## Abstract

Four new species of Desmiphorini are described: Desmiphora (Desmiphora) orozcoi, from Belize; *Estola wappesi*, from Bolivia; *Estola imitatrix*, from Bolivia; and *Gyrpanetes clarkei*, from Bolivia. A key to the species of *Gyrpanetes* is provided, and *Estola wappesi* and *Estola imitatrix* are included in an existing key.

## Introduction

According to Monné (2014), Desmiphorini Thomson, 1860 included 73 genera and 462 species in the Neotropical Region. Recently, Monné and Wappes (2014) described a new genus and species of Desmiphorini. Thus, the total number of genera is now 74, with 463 species. Desmiphora (Desmiphora) Audinet-Serville, 1835, and *Estola* Fairmaire & Germain, 1859 are the genera with the largest number of species, at 67 and 95, respectively. Even so, new species are frequently described in these genera. In this report, we described one new species in Desmiphora (Desmiphora) and two in *Estola* genera.

*Gyrpanetes* Martins & Galileo, 1998, is a small genus with three described species, all of which are known only from Brazil. The new species described herein is the first record of this genus outside of Brazil.

## Material and methods

Photographs were taken with a Canon EOS Rebel T3i DSLR camera, Canon MP-E 65mm f/2.8 1–5× macro lens, controlled by Zerene Stacker AutoMontage software.

The acronyms used in the text are as follows:

ACMT American Coleoptera Museum (James E. Wappes), San Antonio, Texas, USA

MNKM Museo de Historia Natural Noel Kempff Mercado, Santa Cruz, Bolivia

MZSP Museu de Zoologia, Universidade de São Paulo, São Paulo, Brazil

USNM National Museum of Natural History, Washington, DC, USA.

## Taxonomy

### Desmiphora (Desmiphora) orozcoi sp. n.

Taxon classificationAnimaliaColeopteraCerambycidae

http://zoobank.org/4D71CB8E-CD03-4C5D-87B3-1FA9CDBC6C0B

Figs 1 , 2 , 3

#### Description.

Holotype female. Integument black; palpi reddish-brown; basal antennomeres dark-brown, gradually light-brown towards antennomere XI; tibiae and tarsi brown.

Head. Frons transverse; coarsely, abundantly, deeply punctate; pubescence short, yellowish-brown, distinctly not obliterating integument, mixed with long, moderately abundant setae. Area between antennal tubercles and vertex with punctures as on frons; yellowish-brown pubescence distinctly longer, denser than on frons, mixed with sparse small spots of white pubescence, and long, abundant setae. Coronal suture clearly distinct from clypeus to anterior edge of prothorax. Antennal tubercles with pubescence and setae as on frons, but smaller punctures. Area around eyes with narrow band of white pubescence. Area behind upper eye lobes with short, yellowish-brown, moderately sparse pubescence; region behind interconnection area of eyes lobes glabrous; area behind lower eye lobes with short, sparse yellowish-brown pubescence; along area closer to eyes, long, sparse setae. Genae partially striated, transversely with sparse yellowish-brown pubescence, more concentrated in some regions; with long, sparse setae. Gula shiny, glabrous, except for narrow band of yellowish-brown pubescence close to anterior margin. Distance between upper eye lobes equal to 0.75 times length of scape; distance between lower eye lobes equal to 1.25 times length of scape. Antennae as long as 1.2 times elytral length; reaching about distal fifth of elytra; scape, pedicel and antennomeres with long, yellowish-white setae throughout (longer ventrally; shorter on distal antennomeres); antennomeres IV–X with basal ring of whitish pubescence; antennal formula based on antennomere III: scape = 0.77; pedicel = 0.20; IV = 0.92; V = 0.47; VI = 0.46; VII = 0.43; VIII = 0.38; IX = 0.33; X = 0.27; XI = 0.38.

Thorax. Pronotum moderately fine, sparsely punctate (punctures mostly obliterated by pubescence); yellowish-white dense pubescence, forming large “M-like” shape on disc; on each top of “M-like” there is distinct tuft; remaining surface with sparser yellowish pubescence; with long, moderately abundant brown and yellowish-white setae. Sides of prothorax with large, conical tubercle; pubescence yellowish, dense, mixed with long, abundant setae; coarsely, sparsely punctate. Prosternum and prosternal process with short, moderately sparse, yellowish pubescence, mixed by long, sparse setae. Metasternum laterally with yellowish pubescence (white depending on light), centrally glabrous. Scutellum glabrous on central base, laterally with yellowish-white pubescence. Elytra. Moderately coarsely, sparsely punctate on basal third (laterally denser), gradually finer towards apex; pubescence yellowish-brown (distinctly more abundant on basal third and near apex) and white (distinctly more abundant on basal two-thirds of distal half), not forming erect tufts; with long, abundant dark-brown and yellow setae; apex truncate, with outer angle rounded. Legs. Femora with yellowish-brown pubescence, absent on some areas; with long setae. Tibiae and tarsi with long setae.

Abdomen. Urosternites with yellowish pubescence, laterally denser (mainly on urosternite I); distal margin of urosternites I–IV with fringe of yellow pubescence; urosternites II–IV laterally with small spot of whitish pubescence; urosternite V deeply, triangularly depressed at apex.

**Figures 1–6.**
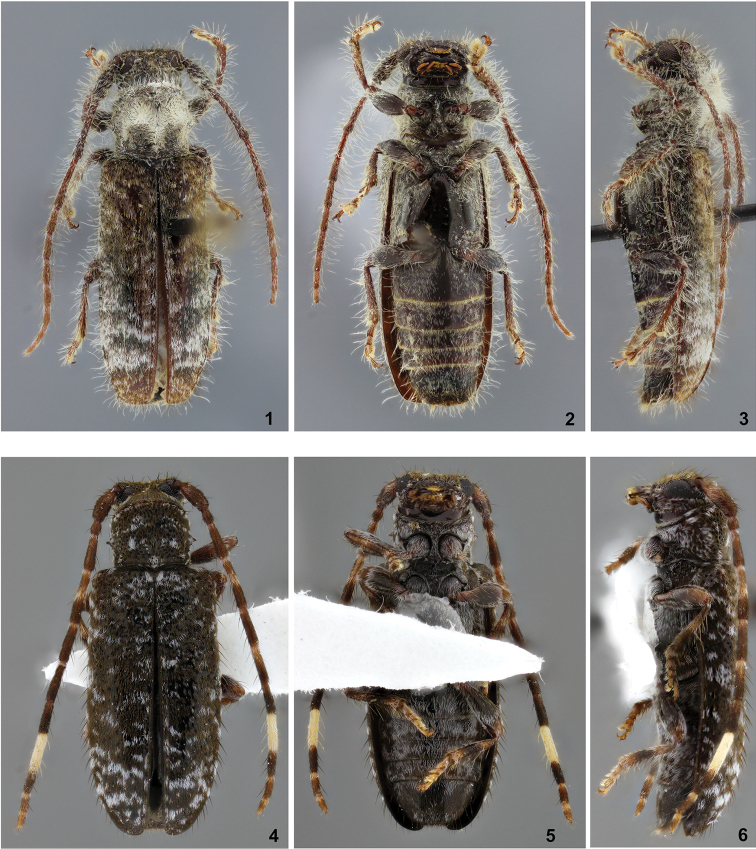
**1–3** Desmiphora (Desmiphora) orozcoi, holotype female: **1** Dorsal habitus **2** Ventral habitus; **3** Lateral habitus. **4–6** *Estola wappesi*, holotype female: **4** Dorsal habitus **5** Ventral habitus **6** Lateral habitus.

#### Dimensions in mm

**(female).** Total length, 9.6; length of prothorax at center, 2.2; greatest width of prothorax (between apices of tubercles), 2.6; anterior width of prothorax, 1.9; posterior width of prothorax, 2.0; humeral width, 2.8; elytral length, 6.8.

#### Type material.

Holotype female, BELIZE, *Cayo District*: Las Cuevas Research Station (580m; 16°43.971'N, 88°59.196'W), 1–4.VI.2008, Ratcliffe, Cave, Jameson and Orozco col. (USNM).

#### Etymology.

The species is named after Jesus Orozco (University of Nebraska State Museum, Nebraska, USA), one of the collectors of the holotype of the new species.

#### Remarks.

Desmiphora (Desmiphora) orozcoi differs from all other species recorded from Central America and the Caribbean due to the absence of erect tufts of pubescence on elytra. The new species is somewhat similar to Desmiphora (Desmiphora) intonsa (Germar, 1824), but differs as follows: elytra do not have tufts of pubescence (present in Desmiphora (Desmiphora) intonsa); elytral apex without dark area, distinctly contrasting with all other regions of the surface (present in Desmiphora (Desmiphora) intonsa); elytral punctures on basal half not distinctly coarse (very coarse in Desmiphora (Desmiphora) intonsa). It can be separated from Desmiphora (Desmiphora) niveocincta (Lane, 1959), by the presence of light pubescence on center basal half of pronotum, by the absence of dense spots with white pubescence on elytra, and by the dark legs. In Desmiphora (Desmiphora) niveocincta there is no light pubescence on the basal center half of the pronotum, the elytra have dense spots with white pubescence (the middle one forming a decumbent tuft), and the legs are pale.

The key by Breuning (1974) is based on many misidentifications, therefore it is not possible to modify it to include this species without a full revision.

### Estola wappesi sp. n.

Taxon classificationAnimaliaColeopteraCerambycidae

http://zoobank.org/B82CAF00-2F62-4ED8-8FA9-D11D592569D6

Figs 4 , 5 , 6

#### Description.

Holotype female. Integument dark-brown, almost black; palpi brown; antennomeres III, V–VII, IX–XI with reddish-brown basal ring; basal two-thirds of antennomere IV reddish-brown, dorsally interrupted by incomplete brown ring; antennomere VIII totally yellowish-white; tibiae brown on about basal half; metatarsi mostly reddish-brown.

Head. Frons transverse; coarsely, abundantly punctate; pubescence brown, moderately dense, not obliterating integument, mixed with small, sparse spots of white pubescence; with long, moderately abundant setae. Area between antennal tubercles and vertex with punctures, pubescence and setae as on frons. Coronal suture distinct from clypeus to level of posterior margin of eyes. Antennal tubercles elevated; finely punctate; pubescence and setae as on frons. Area behind eyes moderately coarsely punctate; pubescence white close to the eyes, brown towards prothorax; with some long setae. Genae with whitish pubescence, more brownish towards frons. Gula shiny, glabrous, except for narrow band of brown pubescence close to anterior margin. Lower eye lobes longer than twice length of genae; distance between upper eye lobes equal to 0.5 times length of scape; distance between lower eye lobes equal to length of scape. Antennae as long as 1.4 times elytral length; reaching elytral apex at about middle of antennomere X; light areas of antennomeres with yellowish-white pubescence; scape and pedicel with sparse, long setae throughout; antennomeres III–X ventrally with sparse, long setae; antennal formula based on antennomere III: scape = 1.20; pedicel = 0.40; IV = 1.52; V = 1.08; VI = 1.04; VII = 0.96; VIII = 1.12; IX = 0.64; X = 0.56; XI = 0.52.

Thorax. Pronotum coarsely, deeply, abundantly punctate; disc with three distinct gibbosities, two sub-rounded, placed antero-laterally, another elongate, placed center-basally; pubescence brown, mixed with spots of white pubescence; with long, sparse setae. Sides of prothorax with acute, distinct tubercle about middle; pubescence brownish, mixed with whitish pubescence; coarsely, abundantly punctate. Prosternum, coarsely, moderately abundantly punctate; pubescence mostly brownish. Prosternal process with brownish pubescence. Metasternum laterally moderately coarsely, sparsely punctate; pubescence yellowish-brown, not obliterating integument. Scutellum centrally with brown pubescence, laterally with white pubescence. Elytra. Coarsely, densely, deeply punctate on basal two-thirds, sparser, finer on apical third; pubescence brown, mixed with spots of white pubescence, more abundant on distal third, forming irregular, transverse bands; with long, moderately abundant setae; apices individually rounded. Legs. Pubescence yellowish-brown, more whitish in some areas.

Abdomen. Urosternites with yellowish-brown pubescence, not obliterating integument.

Variability. Coronal suture inconspicuous between clypeus and antennal tubercles; pubescence behind eyes mostly whitish; antennae in male as long as 1.6 times elytral length; elytral spots of white pubescence somewhat variable in amount and distribution.

#### Dimensions in mm

**(male/female holotype).** Total length, 4.90–5.20/5.50; length of prothorax at center, 1.00–1.10/1.10; greatest width of prothorax (between apices of tubercles), 1.20–1.40/1.40; anterior width of prothorax, 1.05–1.10/1.15; posterior width of prothorax, 1.05–1.10/1.15; humeral width, 1.60–1.60/1.85; elytral length, 3.60–3.65/4.10.

#### Type material.

Holotype female, BOLIVIA, *Santa Cruz*: Refugio Los Volcanes (4 km N Bermejo; 18°06'S, 63°36'W; 1045–1350 m), 11–17.XII.2012, Wappes and Skillman col. (MNKM). Paratypes – 2 males, same data as holotype (MZSP, ACMT); 1 male, same data as holotype, except for: 1000 m, 16–21.X.2007, J. Wappes & A. Cline col. (ACMT); Chaco above Achira (Florida province; Vicoquin Area; 18°07'S / 63°47'W; 1730 m), 1 male, 1 female, 22–25.I.2007, Wappes & Lingafelter col. (USNM, male; ACMT, female).

#### Etymology.

The species is named after James E. Wappes (ACMT), for his friendship and constant help with the specimens studied.

#### Remarks.

*Estola wappesi* differs from *Estola boliviana* Breuning, 1940, as follows: lower eye lobes longer than twice the length of genae; antennomere III mostly dark; elytra with areas of white pubescence. In *Estola boliviana* the lower eye lobes are shorter than twice the length of the genae, the antennomere III is whitish-yellow, and the elytra do not have areas with white pubescence. It differs from *Estola strandiella* Breuning, 1942, mainly by the dark elytra, slightly attenuate towards apex (reddish-brown and more attenuated towards the apex in *Estola strandiella*). *Estola wappesi* differs from *Estola longeantennata* Breuning, 1940, by the darkened elytra, by the light pubescence of elytra that is more abundant on the distal half, and by the antennomere VIII that is totally whitish-yellow. In *Estola longeantennata* the elytra is reddish-brown, the light pubescence on elytra is abundant throughout, and the antennomere VIII is whitish-yellow only on basal third.

*Estola wappesi* can be included in the alternative of couplet “52”, from Breuning (1974) (translated):

**Table:** 

52	Lateral spine of prothorax very thin and sharp	**52’**
–	Lateral spine of prothorax conical	**53**
52’	Light pubescence of elytra abundant throughout; antennomere VIII whitish-yellow only at basal third; elytra reddish-brown	***Estola longeantennata* Breuning, 1940**
–	Light pubescence of elytra more abundant on distal half; antennomere VIII totally whitish-yellow; elytra dark-brown	***Estola wappesi* sp. n.**

### Estola imitatrix sp. n.

Taxon classificationAnimaliaColeopteraCerambycidae

http://zoobank.org/9A488BEB-5972-4796-B8D3-2A1AC130CF05

Figs 7 , 8 , 9

#### Description.

Holotype female. Integument black, except for: antennomere III reddish on basal third; antennomeres IV and VI reddish on basal two-thirds; antennomere V reddish on a narrow ring at base; tibiae with wide reddish ring on basal half, close to middle, and another covering approximately entire distal third; protarsomeres I–II mostly reddish; meso- and metatarsomeres I–III mostly reddish.

Head. Frons transverse; coarsely, moderately abundantly punctate; pubescence yellowish-brown, centrally mixed with white pubescence; with long, moderately abundant setae. Vertex moderately coarsely, abundantly punctate (punctures mostly obliterated by pubescence); pubescence yellowish-brown, mixed with whitish pubescence, more distinctly on central tuft; with long, thick setae. Coronal suture marked from clypeus to anterior edge of prothorax, but mostly obliterated by pubescence. Antennal tubercles with yellowish-brown pubescence. Area behind eyes with yellowish-brown pubescence, more whitish, slightly denser closer to eyes; with long, yellowish setae behind lower eye lobes, mainly near eyes. Genae with yellowish-brown pubescence, mixed with sparse long setae. Gula glabrous, with very fine, sparse punctures towards prothorax; punctures slightly more distinct and sparse short setae present towards anterior edge; area close to anterior edge with band of yellowish-brown pubescence. Lower eye lobes about 2.5 times longer than genae; distance between upper eye lobes equal to 0.55 times length of scape; distance between lower eye lobes equal to length of scape. Length of antennae from base of scape to apex of antennomere VI equal to 0.90 times elytral length (lacking antennomeres VII-XI); scape and pedicel with sparse, long setae throughout; antennomeres III–VI ventrally with sparse, long setae; antennal formula based on antennomere III: scape = 1.23; pedicel = 0.42; IV = 1.34; V = 1.11; VI = 1.00.

Thorax. Prothorax transverse. Pronotum coarsely, abundantly punctate (punctures partially obliterated by pubescence); pubescence yellowish-brown, moderately dense, on central triangular area, wide oblique band with white, dense pubescence on basal half, remaining surface with white pubescence, mixed with yellowish-brown pubescence; with long, thick, sparse setae (mainly anteriorly). Sides of prothorax with short, conical tubercle slightly before middle; coarsely, abundantly punctate; pubescence on tubercle white; remaining surface with yellowish-brown pubescence, denser towards ventral side of thorax, mixed with white pubescence on some areas. Ventral side of thorax with yellowish-brown pubescence, mixed with long setae (mainly centrally on prosternum and mesosternum). Scutellum centrally with brown pubescence, laterally with yellowish-brown pubescence. Elytra. Coarsely, densely, deeply punctate on basal half, sparser on distal half; pubescence, yellowish-brown, mixed with white pubescence; the latter forming a basal band, following along suture and connected with wide, transverse, somewhat distinct band slightly after middle, not reaching lateral margin; with long, thick, moderately abundant setae throughout; apex individually rounded. Legs. With long, yellow, moderately abundant setae.

Abdomen. Urosternites with yellowish-brown pubescence.

**Figures 7–12.**
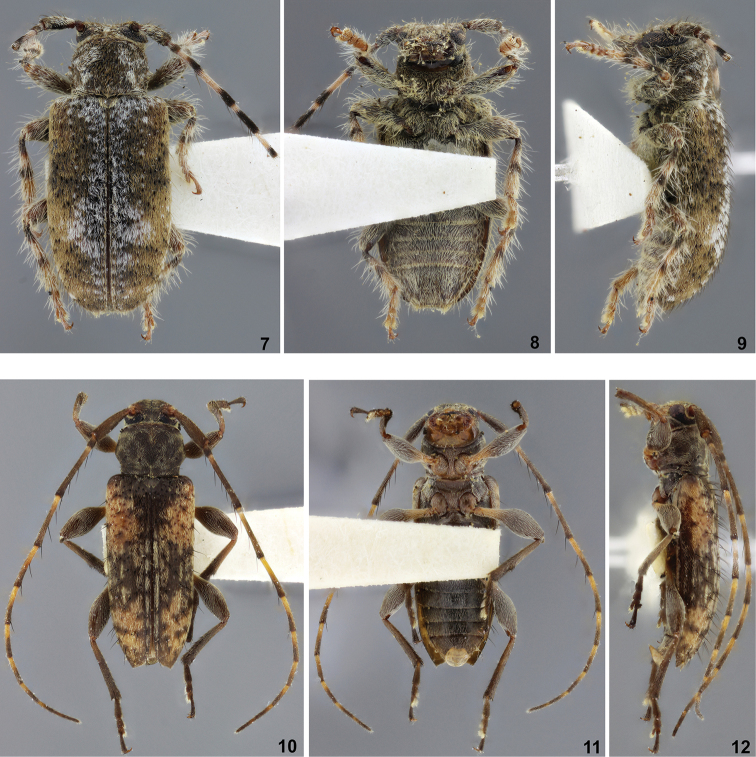
**7–9** *Estola imitatrix*, holotype female: **7** Dorsal habitus **8** Ventral habitus **9** Lateral habitus **10–12** *Gyrpanetes clarkei*, holotype male: **10** Dorsal habitus **11** Ventral habitus **12** Lateral habitus.

#### Dimensions in mm

**(female).** Total length, 5.75; length of prothorax at center, 1.20; greatest width of prothorax (between apices of tubercles), 1.65; anterior width of prothorax, 1.40; posterior width of prothorax, 1.45; humeral width, 2.30; elytral length, 4.30.

#### Type material.

Holotype female from BOLIVIA, *Tarija*: 300m S Palo Marcado (21°28'S, 63°08'W; *ca.* 300m; edge of Pilcomayo River; dry Chaco Forest; beaten dry *Acacia* tree), 12.XII.2007, R. Clarke & S. Zamalloa col. (MNKM).

#### Etymology.

Latin, *imitatrix* = a female imitator; allusive to similar appearance to *Estola basiflava* Breuning, 1943.

#### Remarks.

*Estola imitatrix* differs from *Estola densepunctata* Breuning, 1940, and *Estola basiflava* as follows: body is wider; antennomere III, distinctly shorter than scape; antennomere III, light only on basal third; and pronotum has bands of white pubescence. In both species the body is slightly narrower, the antennomere III is about as long as the scape (sometimes slightly shorter) and dark, at most, on distal third (32 specimens of *Estola densepunctata*, and 2 of *Estola basiflava* were examined), and the pronotum has no bands with white pubescence. It differs from *Estola compacta* Breuning, 1940, by the white pubescence on the base of elytra, prolonged along the suture (yellowish and not prolonged in *Estola compacta*), and by the antennomere III being widely-ringed with black (entirely yellowish in *Estola compacta*). *Estola imitatrix* can be separated from *Estola fuscomarmorata* Breuning, 1940, by the body being shorter and wider (narrower and more elongated in *Estola fuscomarmorata*), by the antennomere III being distinctly shorter than IV (about the same length in *Estola fuscomarmorata*), and by the antennomere III being ringed with black (entirely yellowish in *Estola fuscomarmorata*).

*Estola imitatrix* can be included in the alternative of couplet “6”, from Breuning (1974) (translated; modified):

**Table:** 

6’	Pronotum and elytra distinctly spotted with white pubescence	***Estola imitatrix* sp. n.**
–	Pronotum and elytra not spotted with white pubescence	**6**
6(6’)	Only the extreme base of the elytra with pale yellow pubescence	***Estola basiflava* Breuning, 1943**
–	Basal fourth of elytra with pale yellow pubescence	***Estola flavobasalis* Breuning, 1940**

### Gyrpanetes clarkei sp. n.

Taxon classificationAnimaliaColeopteraCerambycidae

http://zoobank.org/C2257CD2-E942-4922-8504-5B44E706849D

Figs 10 , 11 , 12

#### Description.

Holotype male. Integument dark-brown, almost black on some areas, except for: base of antennomere III–X (about basal half on antennomeres V–IX), gula, and peduncle of femora reddish-brown; one large, irregular, orangish area on each half of elytron.

Head. Frons transverse, fine, densely punctate; pubescence yellowish-brown, abundant, but not obliterating integument, denser, yellowish close to lower eye lobes. Antennal tubercles moderately elevate; sculpture as on frons; pubescence yellowish-brown towards inner side, more yellowish close to eye. Coronal suture distinct from clypeus to anterior edge of prothorax. Vertex with sculpture and pubescence as on frons. Area behind eyes with band of yellowish pubescence close to eyes, gradually glabrous towards anterior edge of prothorax. Genae with yellowish pubescence (more whitish depending on angle of light). Gula shiny, glabrous, except for band of yellowish pubescence close to anterior margin. Distance between upper eye lobes equal to 0.35 times length of scape; distance between lower eye lobes equal to 0.65 times length of scape. Antennae as long as 2.0 times elytral length; reaching elytral apex about base of antennomere VII; pedicel and antennomeres III–VI with long, dark setae on ventral side (sparser towards antennomere VI); light area of antennomeres with yellowish pubescence; antennal formula based on antennomere III: scape = 0.78; pedicel = 0.19; IV = 0.89; V = 0.73; VI = 0.65; VII = 0.56; VIII = 0.51; IX = 0.46; X = 0.40; XI = 0.38.

Thorax. Pronotum fine, densely punctate; disc with whitish-yellow pubescence (more whitish depending on angle of incision of light), shorter and slightly sparser on area forming “M-like shape” (less distinct depending on the viewing angle). Sides of prothorax with whitish-yellow pubescence, shorter and slightly sparser on central sub-rounded area; sculpture as on pronotum. Ventral side of thorax with yellowish-brown pubescence (more whitish depending on angle of light), not obliterating integument. Elytra. Center basal region without crest; sparse, coarsely punctate on basal half (denser around scutellum), distinctly sparser on distal half; with thick, moderately abundant, very long, dark setae throughout; pubescence whitish-yellow (more whitish depending on angle of incision of light), whitish and yellowish-brown, forming irregular drawing; around some punctures of distal half, pubescence forming ocellar macula; with two slightly distinct longitudinal dashed lines with whitish pubescence, more distinct around middle; apex obliquely truncate, with sutural angle rounded. Legs. Pubescence as on urosternites.

Abdomen. Pubescence on urosternites whitish-yellow (more whitish depending on angle of light), not obliterating integument

Variability. Gula and peduncle of femora brown.

#### Dimensions in mm

**(male).** Total length, 4.10–4.35; length of prothorax at center, 0.80–0.85; greatest width of prothorax, 1.05–1.10; anterior width of prothorax, 0.85–0.90; posterior width of prothorax, 0.85–0.90; humeral width, 1.25–1.35; elytral length, 3.00–3.10. The smallest dimensions are those of the holotype.

#### Type material.

Holotype and paratype male from BOLIVIA, *Tarija*: 24 km W Villamontes (21°21'S, 63°37'W; *ca.* 800 m; Valley Rio Isiri; Submontane Chaco Forest), 11.I.2008, R. Clarke & S. Zamalloa col. (holotype, MNKM; paratype, MZSP).

#### Etymology.

The species is named after Robin O. S. Clarke, one of the collectors of the type specimens.

#### Remarks.

*Gyrpanetes clarkei* differs from *Gyrpanetes oriba* Galileo & Martins, 2003, as follows: antennomere III is reddish only at base; pronotal pubescence forming “M-like” macula; elytral dashed lines with whitish pubescence partially distinct; elytral pubescence has areas distinctly exposing larger and more distinct integument (dark areas). In *Gyrpanetes oriba* the antennomere III is dark only at the apex, the pronotal pubescence does not form “M-like” macula, the elytral dashed lines are distinct from base to near apex, and the elytral pubescence is more distinctly whitish throughout.

Martins and Galileo (1998) included a key to separate the two species of the genus. It is necessary to provide a new key to include the four species of the genus:

**Table:** 

1	Center basal crest of elytra distinctly elevated. Brazil (Paraná)	***Gyrpanetes pukuaba* Martins & Galileo, 1998**
–	Center basal crest of elytra not elevated	**2**
2(1)	Elytra with very sparse long setae. Brazil (Espírito Santo)	***Gyrpanetes cacapira* Martins & Galileo, 1998**
–	Elytra with distinct long setae throughout	**3**
3(2)	Annnomere III mostly reddish; pronotum without “M-like” macula; elytral dashed lines distinct from base to near apex. Brazil (Espírito Santo)	***Gyrpanetes oriba* Galileo & Martins, 2003**
–	Antennomere III mostly dark; pronotum with “M-like” macula; elytral dashed lines slightly distinct (more distinct around middle). Bolivia	***Gyrpanetes clarkei* sp. n.**

## Supplementary Material

XML Treatment for Desmiphora (Desmiphora) orozcoi

XML Treatment for Estola wappesi

XML Treatment for Estola imitatrix

XML Treatment for Gyrpanetes clarkei
